# Coronary artery calcium quantification technique using dual energy material decomposition: a simulation study

**DOI:** 10.1007/s10554-024-03124-9

**Published:** 2024-06-21

**Authors:** Dale Black, Tejus Singh, Sabee Molloi

**Affiliations:** Department of Radiological Sciences, University of California, Medical Sciences I, B-140, Irvine, CA 92697 USA

**Keywords:** Calcium scoring, Material decomposition, Volume fraction calcium mass, Computed tomography, Agatston scoring

## Abstract

Coronary artery calcification is a significant predictor of cardiovascular disease, with current detection methods like Agatston scoring having limitations in sensitivity. This study aimed to evaluate the effectiveness of a novel CAC quantification method using dual-energy material decomposition, particularly its ability to detect low-density calcium and microcalcifications. A simulation study was conducted comparing the dual-energy material decomposition technique against the established Agatston scoring method and the newer volume fraction calcium mass technique. Detection accuracy and calcium mass measurement were the primary evaluation metrics. The dual-energy material decomposition technique demonstrated fewer false negatives than both Agatston scoring and volume fraction calcium mass, indicating higher sensitivity. In low-density phantom measurements, material decomposition resulted in only 7.41% false-negative (CAC = 0) measurements compared to 83.95% for Agatston scoring. For high-density phantoms, false negatives were removed (0.0%) compared to 20.99% in Agatston scoring. The dual-energy material decomposition technique presents a more sensitive and reliable method for CAC quantification.

## Introduction

Coronary artery calcification (CAC) serves as an important atherosclerotic marker and a primary predictor of cardiovascular disease, which is currently the predominant cause of mortality in the United States [1]. Computed tomography (CT) is a non-invasive technique routinely utilized for quantifying CAC [2].

Among the various CAC scoring methods, Agatston scoring [3] is the most prevalent. It has demonstrated efficacy in predicting major adverse cardiac events (MACE) [4, 5]. However, despite its wide application, the Agatston scoring approach is subject to several shortcomings, including a lack of repeatability, potential inaccuracy, and diminished sensitivity to microcalcifications [6–9]. These limitations are, in part, attributable to the arbitrary thresholding involved in the Agatston scoring process. This method omits all voxels with values below a typical threshold of 130 Hounsfield units, thereby excluding potential low-density and micro-calcifications [8]. Moreover, score variations across different vendors and scanners further add to the inherent arbitrariness of the method [10].

Dual-energy CT is an emerging technology that has been shown to improve the visualization of calcified plaque constituents and enhance overall plaque assessment [11]. The principle of dual-energy CT is rooted in the differential mass attenuation coefficients exhibited by diverse tissues when exposed to X-rays of varying energies [12]. Consequently, dual-energy CT facilitates a material-specific analysis of coronary CT imaging, presenting the potential to quantify calcium mass in dual-energy CT scans directly.

Despite research exploring material decomposition in multi-energy CT for CAC detection, no studies to date have investigated direct physical quantification of CAC [13, 14]. Moreover, comparative studies between material decomposition-based methods and the standard Agatston score are absent.

In this study, we introduce a calcium mass quantification approach for CAC, based on dual-energy material decomposition, with the specific aim of enhancing detectability compared to current CAC quantification techniques. We compare this method with the ground truth calcium mass using simulated calcification phantoms. Further, we draw a comparison between the established Agatston scoring method, a recently proposed single-energy CAC quantification technique called volume fraction calcium mass [15], and our proposed method.

## Methods

### Simulation

The simulation study was designed to match the scanning parameters of the 320-slice CT scanner (Canon Aquilion One, Canon America Medical Systems, Tustin, CA), as previously reported [16]. The X-ray spectrum was created with an interpolating polynomial model [17]. The linear attenuation coefficients were made from their chemical composition. These linear attenuation coefficients were derived from the chemical composition of calcium hydroxyapatite and the energy-dependent mass attenuation coefficients obtained from the National Institute of Standards and Technology database. Poisson noise was added to simulate quantum noise. The simulation did not include Compton scatter, the dominant attenuation mechanism in CT imaging due to the interaction of free electrons with the incoming X-ray, but beam hardening was included. All scans utilized filtered back projection for reconstruction. Previous studies describe the simulation in detail [15], and Table 1 summarizes the important details.

**Fig. 1:**
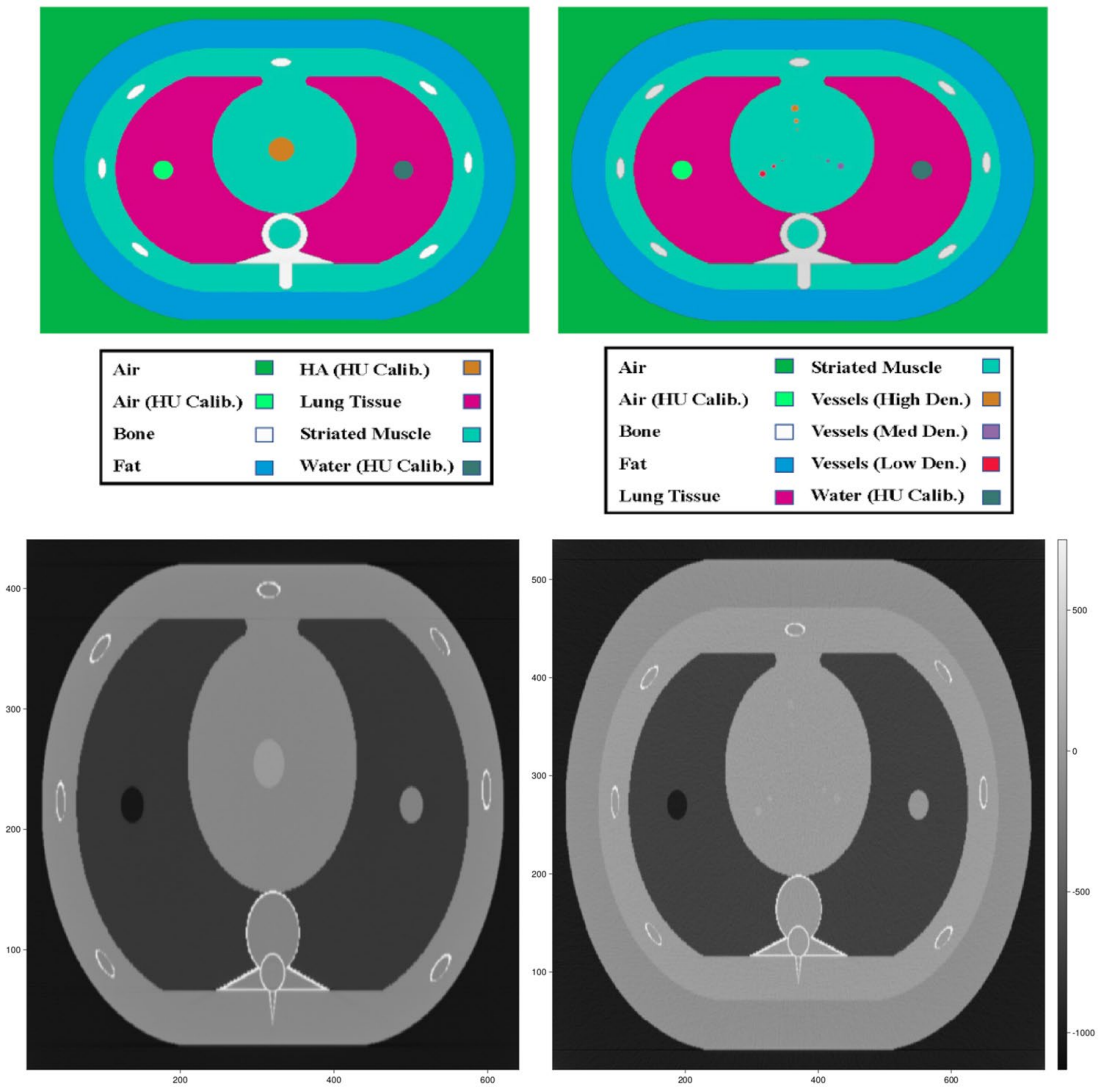
Shows a sketch of the simulated phantoms. The top row shows the phantoms with the colors highlighting the different materials in the simulated phantoms. The bottom row shows slices of the actual phantom images. The bottom left image shows a slice of a small sized calibration phantom with a rod density of 100 mgHAcm-3 at 80 kV. The bottom right image shows a slice of a medium sized low-density measurement phantom with calcification inserts (52, 59, and 73 mgHAcm^−3^) at 135 kV

Figure 1 illustrates the simulated materials and geometries, which were based on the QRM-D100 Calcium Scoring Phantom insert as part of the QRM-20103 Thorax Phantom set (QRM GmbH, Möhrendorf, Germany). The QRM-D100 phantom insert contains nine cylindrical calcification inserts with varying sizes and calcium hydroxyapatite densities, as well as two larger calibration inserts (water-equivalent and calcium hydroxyapatite material). All calcium quantification measurements were repeated across the corresponding tube voltages (80/135 kV for material decomposition, 120 kV for Agatston and volume fraction methods), patient sizes (small, medium, large), calcium insert sizes (1, 3, 5 mm), and calcium insert densities (15–780 mgHAcm^−3^).

Segmentation of regions of interest is an important step in calcium measurement. For this study, automatic segmentation was implemented based on the work of Praagh et al. [18] and adapted to the simulated phantoms. This approach effectively segments calcium inserts based on the known phantom geometry, thus eliminating the need for manual intervention.

**Table 1:** Summary of simulation parameters

Parameter	Description
CT scanner	Canon aquilion one
Phantom type	QRM thorax
Reconstruction	FBP
Phantom dimensions small (cm^2^)	30 x 20 (640 x 440 pixels)
Phantom dimensions medium (cm^2^)	35 x 25 (740 x 540 pixels)
Phantom dimensions large (cm^2^)	40 x 30 (840 x 640 pixels)
Fat ring composition	20:80 water/lipid mixture
Calcification inserts length (mm)	1.5
Calcification inserts diameters (mm)	1, 3, 5
Insert densities low (mgHAcm^−3^)	15–73
Insert densities normal (mgHAcm^−3^)	110–780
Tube voltages dual energy (kV)	80/135
Tube voltages single energy (kV)	120
Exposure values small (mR)	0.9
Exposure values medium (mR)	2.0
Exposure values large (mR)	5.4
Detector element width (mm)	0.5
Detector thickness (mm)	0.5

### Agatston scoring

Agatston scoring is the most widely used method for quantifying coronary artery calcification (CAC) in clinical practice [3]. This method relies on the identification of high-density regions within the coronary arteries, typically using a threshold of 130 Hounsfield units (HU) or higher. The Agatston score is calculated by multiplying the area of each high-density region by a weighting factor based on the maximum density of the region. The weighting factors are assigned as follows: 1 for regions with a maximum density of 130–199 HU, 2 for 200–299 HU, 3 for 300–399 HU, and 4 for regions with a maximum density of 400 HU or higher. The total Agatston score is the sum of the weighted scores for all high-density regions identified within the coronary arteries.

### Volume fraction calcium mass

The volume fraction calcium mass technique is a method for quantifying the amount of calcium within a voxel. It has been demonstrated to be a more accurate approach for single-energy CAC quantification when compared to the conventional Agatston scoring method [15]. This technique has been previously elaborated upon in detail [15] and Eq. (1) outlines the steps involved in quantifying calcium using the volume fraction calcium mass technique:

1 $$\begin{aligned} k_i&= \frac{i - S_{Obj}}{S_{Obj} - S_{Bkg}} \nonumber \\ K&= \sum _i k_i \nonumber \\ V_{Obj}&= (K) (V_{ROI}) \nonumber \\ M_{Obj}&= (V_{Obj}) (\rho _{S_{Obj}}) \end{aligned}$$

Here, $S_{Obj}$ is the signal of the calcium from the calibration rod, $S_{Bkg}$ is the signal of pure background, and *i* is the raw signal of the voxel. Therefore, $k_i$ is then the percentage of calcium within an individual voxel, *K* is the total calcium percentage within a region of interest, $V_{ROI}$ is the volume of the region of interest, $V_{Obj}$ is the volume of the calcium, $\rho _{S_{Obj}}$ is the density of the calcium used in the calibration, and $M_{Obj}$ is the mass of the unknown calcium.

### Material decomposition

Dual-energy material decomposition is a fully automated technique that enables the quantification of material compositions within a CT scan without the need for manual correction. The method involves two main steps: (1) calibration of the system matrix coefficients using a phantom with known material compositions, and (2) application of the calibrated coefficients to decompose unknown material compositions in the regions of interest [11, 12, 16, 19, 20].

Recently, Ding et al. proposed an approach to characterize coronary plaques using image-domain-based dual-energy material decomposition [16]. Building upon this approach, the present study aims to compare the accuracy, sensitivity, and specificity of this material decomposition technique for CAC quantification.

The calibration process involves scanning a phantom with known material compositions at low and high energy levels (80 kVp and 135 kVp in this study). The phantom contains inserts with varying concentrations of the basis materials (e.g., water and calcium in this study). The CT numbers (in Hounsfield units) of these inserts are measured, and the known material concentrations are substituted into a system of non-linear equations (Eq. 2). A least-squares fitting algorithm, such as the Levenberg-Marquardt algorithm [21], is then used to solve for the calibration coefficients ($p_0$, $p_1$, $p_2$, $p_3$, $p_4$, $p_5$, $p_6$, and $p_7$) for each material. These coefficients are specific to the CT scanner and acquisition parameters used, and they remain valid as long as the scanner and parameters are not changed.

2 $$\begin{aligned} f = \frac{p_0 + p_1 S^L + p_2 S^H + p_3 (S^L)^2 + p_4 S^L S^H + p_5 (S^H)^2}{1 + p_6 S^L + p_7 S^H} \end{aligned}$$

Once the coefficients are determined, Eq. 2 is then used to predict the density (*f*) given the signal from a voxel from the low and high-energy images ($S^L$, $S^H$). The density of the voxel is then multiplied by the volume of the voxel ($V_{ROI}$) to calculate the mass of the calcification ($M_{Obj}$).

3 $$\begin{aligned} M_{Obj} = f \times V_{ROI} \end{aligned}$$

The regions of interest can be automatically segmented using previously validated methods, such as atlas-based or deep learning-based approaches. This automation eliminates the need for manual intervention, making the method more efficient and less prone to human error. For this study, the fully automated quantification method was adapted for use in automatically segmenting the regions of interest [18].

The computation time for the dual-energy material decomposition method is relatively short. The calibration step, which involves solving a system of non-linear equations using least-squares fitting, takes approximately 2.315 ms on a standard laptop computer. Once the calibration coefficients are determined, the decomposition of unknown material compositions within the region of interests is performed on a voxel-by-voxel basis, taking approximately 293.333 ns for a typical CT scan with 320 slices.

This study utilized a simulated calibration phantom that mimics the commercially available Gammex 472 phantom (Gammex Inc., Middleton, WI, USA). The calcium calibration rods consisted of eight different densities (0, 50, 100, 200, 300, 400, 500, 600 mgHAcm^−3^).

### Statistical analysis

All calcium scoring computations and analyses were executed in the Julia programming language [22]. Root mean square error (RMSE) and root mean square deviation (RMSD) were calculated for all linear regression measurements to evaluate accuracy (RMSE) and precision (RMSD). Equation 4 details the computation of RMSE and RMSD. In this equation, *N* is the total number of data points, $\hat{y}$ represents the calculated calcium masses and *y* denotes the ground truth calcium masses (for RMSE) or the linear regression-based calcium masses (for RMSD), which are computed based on the calculated calcium masses.

4 $$\begin{aligned} RMS = \sqrt{\frac{\sum |y - \hat{y}|^2}{N}} \end{aligned}$$

False-negative and false-positive percentages were also computed for each calcium quantification technique. For the Agatston scoring method, a false-negative (CAC = 0) score is assigned whenever a region of interest, known to contain some amount of calcium, yields a CAC score of precisely zero. As the material decomposition and volume fraction calcium mass methods measure real masses, which can be negative or positive, a false-negative (CAC = 0) score was defined as any calculated mass less than or equal to the mean calcium mass of the pure background (as measured by the respective method), plus 1.5 standard deviations. Similarly, a false-positive (CAC > 0) score was assigned to any mass calculation on pure background that exceeded the mean calcium mass of pure background by more than 1.5 standard deviations. The additional standard deviation in the threshold is intended to prevent false-positive (CAC > 0) scores in regions without calcium. The image analysis was conducted by author, Dale Black, with over five years of medical imaging physics research experience.

## Results

### Detectability

The simulated phantoms were categorized into two groups, low-density and high-density. The low-density group, comprising phantoms with densities ranging from 15 to 73 mgHAcm^−3^, was analyzed separately from the high-density group, which included phantoms with densities from 110 to 780 mgHAcm^−3^.

Out of a total of 81 calcifications, the material decomposition, volume fraction calcium mass, and Agatston scoring methods produced 6, 24, and 68 false-negatives (CAC = 0), respectively, for the low-density phantoms. In the high-density group, these methods yielded 0, 9, and 17 false-negatives (CAC = 0), respectively, out of 81 total calcifications.

**Fig. 2:**
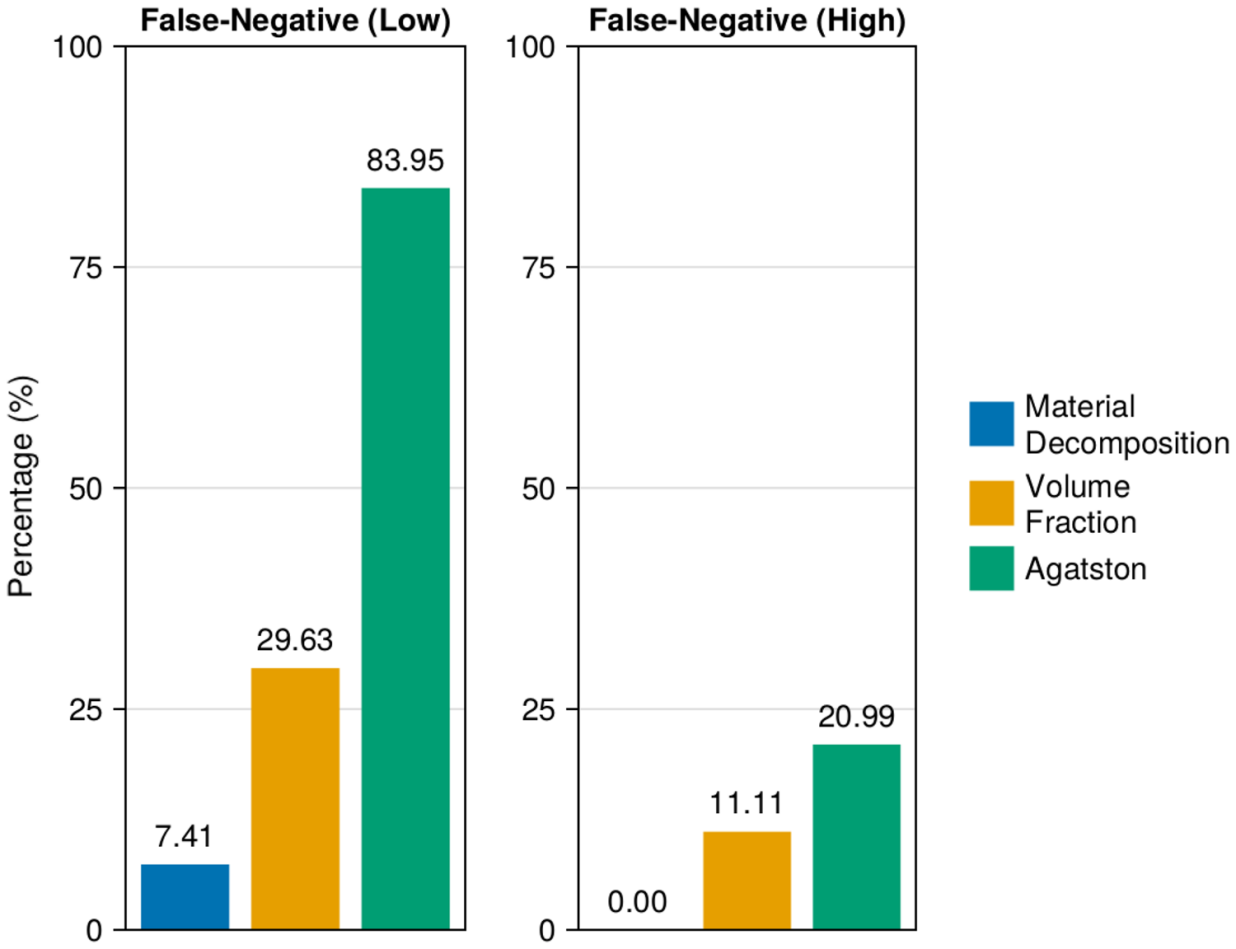
Percentage of false-negative (CAC=0) scores for material decomposition, volume fraction calcium mass, and Agatston scoring techniques. The left column corresponds to the calculations within the low-density (15–73 mgHAcm^−3^) group, and the right column pertains to the high-density group (110–780 mgHAcm^−3^)

Figure 2 presents the percentages of false-negative (CAC = 0) scores generated by all three techniques. In the low-density group, the material decomposition, volume fraction calcium mass, and Agatston scoring methods resulted in 7.41, 29.63, and 83.95% false negative scores (CAC = 0), respectively. In the high-density group, material decomposition yielded no false-negatives (0.00%) and volume fraction calcium mass resulted in 11.11% false-negatives (CAC = 0). The Agatston scoring method had a higher false-negative (CAC = 0) percentage of 20.99% in the high-density regime.

It is important to note that no false-positives (CAC > 0) occurred from any of the scoring techniques in either the low-density or high-density groups, indicating a high specificity for all methods.

### Accuracy

**Fig. 3:**
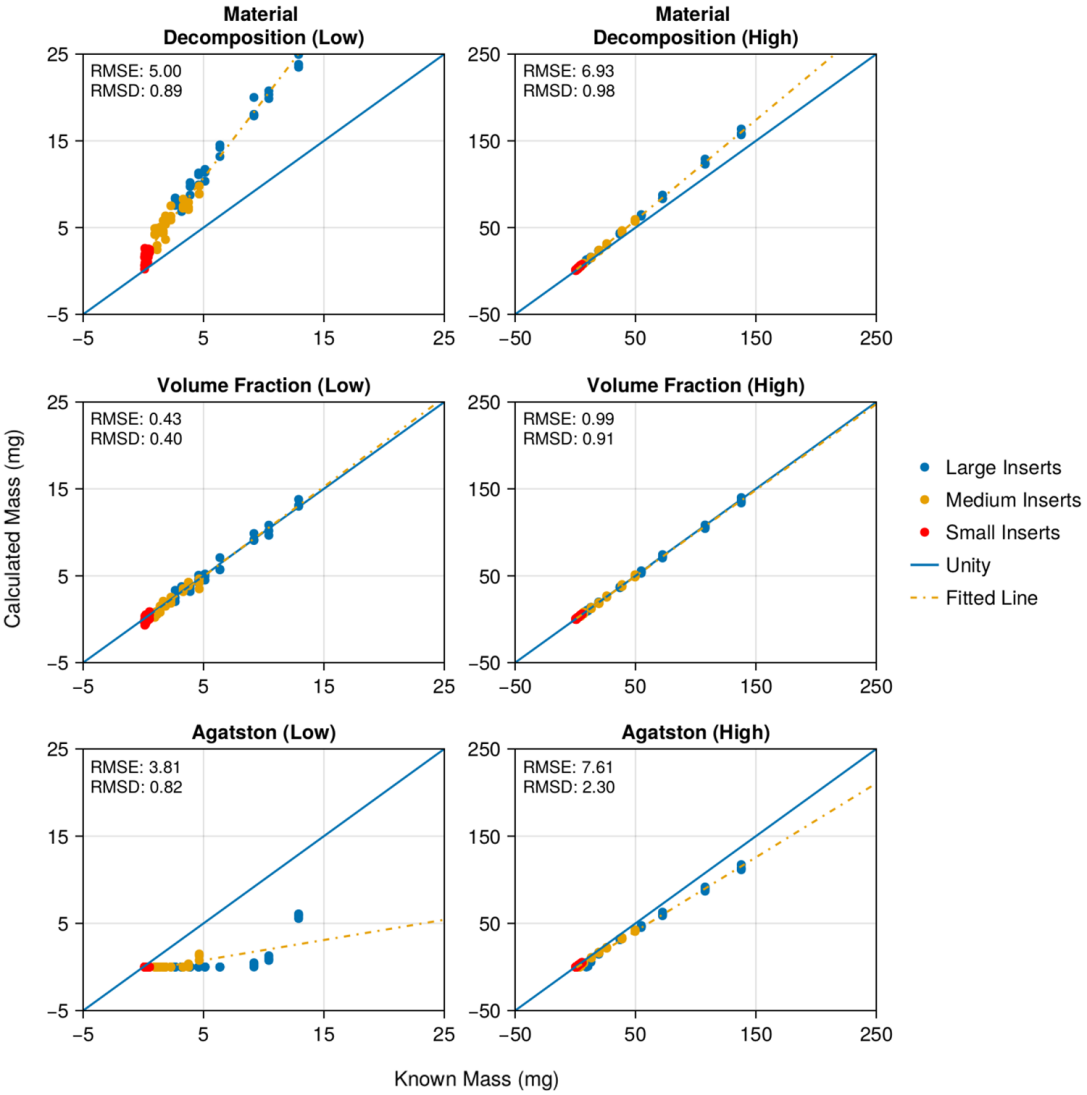
Comparison of measured and known calcium values for three different CAC quantification techniques. The left column corresponds to the calculations within the low-density (15–73 mgHAcm^−3^) group, and the right column pertains to the high-density group (110–780 mgHAcm^−3^)

In both low and high-density groups, the volume fraction calcium mass technique outperformed the material decomposition technique in terms of accuracy. Specifically, for the low-density phantoms, the volume fraction method achieved an RMSE of 0.43 mg, while the material decomposition method resulted in an RMSE of 5.00 mg. Likewise, for the high-density phantoms, the volume fraction method (0.99 mg RMSE) was more accurate than the material decomposition method (6.93 mg RMSE). The Agatston scoring method was found to be the least accurate, with an RMSE of 3.81 mg for low-density (largely due to false-negative scores of zero) and 7.61 mg for high-density phantoms. Figure 3 shows the RMSE and RMSD for all three techniques, with the results for the low-density group presented in the left column and those for the high-density group in the right column.

### Comparison of Methods

Table 2 presents a comparison of the calcium quantification methods across different phantom sizes and insert diameters. The mean, standard deviation (SD), and coefficient of variation (CV) were calculated for each method, averaged over all insert densities. The ground truth values are also included for comparison. The material decomposition method consistently overestimated the calcium mass across all phantom sizes and insert diameters, while the volume fraction method showed a slight underestimation for larger insert diameters and an overestimation for smaller inserts. The Agatston scoring method systematically underestimated the calcium mass across all phantom sizes and insert diameters.

Despite the systematic overestimation, the material decomposition method demonstrated the lowest CVs compared to the other methods, indicating better relative sensitivity. This suggests that the material decomposition method may be more sensitive to detecting calcium, even if the absolute quantification is less accurate, which is demonstrated in Fig. 2. In contrast, the Agatston scoring method showed the highest CVs, indicating lower relative sensitivity and a higher likelihood of false-negative results.

**Table 2:** Comparison of calcium quantification methods across different phantom sizes and insert diameters, including ground truth values, mean, standard deviation, and coefficient of variation, averaged over all insert densities

Method	Insert diameter	Phantom size (mg)			Ground truth (mg)	Mean (mg)	SD (mg)	CV (%)
		Large	Medium	Small				
Material decomposition	Large	36.56	36.67	37.63	28.90	36.95	43.18	116.84
	Medium	13.95	13.90	13.81	10.41	13.89	15.39	110.80
	Small	2.72	2.25	2.15	1.16	2.37	1.74	73.41
Volume fraction	Large	28.22	28.52	29.36	28.90	28.70	38.26	133.30
	Medium	10.21	10.09	10.54	10.41	10.28	13.94	135.64
	Small	1.18	0.97	0.99	1.16	1.05	1.79	170.88
Agatston	Large	20.23	20.52	21.29	28.90	20.68	34.07	164.76
	Medium	7.18	7.18	7.47	10.41	7.28	12.48	171.45
	Small	0.72	0.78	0.75	1.16	0.75	1.50	200.19

Table 3 presents the sensitivity, specificity, positive predictive value (PPV), and negative predictive value (NPV) for each calcium quantification method. These metrics were calculated using the combined data from all phantom sizes and insert densities. The material decomposition method demonstrated the highest sensitivity (100%) and NPV (100%) among the three methods, indicating its superior ability to detect the presence of calcium and correctly identify true negative cases. The Agatston scoring method showed the highest specificity (100%) and PPV (100%), suggesting that it is highly accurate in identifying true positive cases and avoiding false positives. The volume fraction method had sensitivity (91.36%), specificity (88.89%), PPV (89.16%), and NPV (91.14%), indicating a balanced performance across all diagnostic metrics.

**Table 3:** Sensitivity, specificity, positive predictive value (PPV), and negative predictive value (NPV) for each calcium quantification method, calculated using combined data from all phantom sizes and insert densities

Method	Sensitivity (%)	Specificity (%)	PPV (%)	NPV (%)
Agatston	47.53	100.00	100.00	65.59
Material decomposition	100.00	87.04	88.52	100.00
Volume fraction	91.36	88.89	89.16	91.14

## Discussion

This study presents a new method of coronary artery calcification quantification using dual-energy material decomposition, which intends to improve the detection of calcifications and provide a more sensitive measure of CAC. A comparison of this new technique was made against the traditional Agatston scoring method and the newer single-energy CAC quantification technique - volume fraction calcium mass.

The dual-energy material decomposition technique demonstrated fewer false negatives than Agatston scoring and volume fraction calcium mass in the low-density and high-density phantoms. This result emphasizes the potential of this technique for improved detection of low-density calcifications and microcalcifications, which are often missed by Agatston scoring [23]. Notably, none of the methods generated false positives, which shows the specificity of these techniques.

In terms of calcium mass measurement, the volume fraction calcium mass technique was more accurate than the dual-energy material decomposition method for both low and high-density phantom groups. Despite the reduced accuracy of the material decomposition method relative to volume fraction calcium mass, it still outperformed Agatston scoring, especially in the low-density range. Hence, material decomposition-based CAC quantification shows potential as a more reliable and sensitive method for CAC quantification.

This simulation study has certain limitations. A single slice thickness (0.5 mm) and reconstruction technique (filtered back projection) were used, which does not allow for a robust comparison of the techniques under various parameters. However, several studies show good agreement for Agatston scoring among various reconstruction techniques and improved accuracy when using a decreased slice thickness of 0.5 mm [24, 25]. Additionally, the simulation is constrained by the type and number of materials included within potential plaque regions. To align closely with the commercially available physical anthropomorphic phantom (QRM-20103 Thorax, QRM, Mohrendorf, Germany), the simulation background tissue was designated as PMMA, and the inserts comprised various calcium hydroxyapatite densities. In patients, the regions of interest will contain at least three different mixtures—(1) hyper-attenuating blood, (2) tissue, (3) and potential (calcified) plaque. The simulation is restricted to only two mixtures, so the true strengths of these calcium quantification techniques remain unclear. Dual-energy material decomposition can decompose more than two materials [16, 26], so more realistic CT data may further underscore the benefits of dual-energy material decomposition as a CAC quantification technique.

Previous research shows that Agatston scoring systematically underestimates calcium, which is exacerbated in early-stage plaques (low-density) or those affected by motion [6]. Werf et al. have postulated that motion-related blurring may cause low-density calcifications to fall below the 130 HU threshold, consequently reducing the Agatston score and detectability [7]. Our results agree with Tzolos et al., demonstrating that Agatston scoring results in more false-negative (CAC = 0) CAC scores for small and low-density calcifications [8]. The findings of our study agree with these observations, demonstrating that Agatston scoring yielded the most false-negative classifications, especially within the low-density phantom group.

It is important to note that in this study, the dual-energy material decomposition method demonstrated lower accuracy compared to the volume fraction calcium mass technique. This limitation is likely due to the calibration requirements and the simplified nature of the simulated phantoms, which contain only two materials (calcium hydroxyapatite and soft tissue) within each voxel region of interest. In real-world clinical settings, voxels often contain a mixture of multiple materials, and the dual-energy material decomposition technique has the potential to outperform both the Agatston scoring and volume fraction calcium mass methods in terms of detectability and accuracy. To compensate for this limitation and further improve the performance of the dual-energy material decomposition method, future studies should explore more advanced material decomposition techniques, such as the multi-material decomposition technique proposed by [27]. This technique allows for the decomposition of more than three materials (N>3) and does not require a calibration step, potentially providing more consistent and accurate measurements in scenarios where multiple materials are present within a single voxel.

Radiation dose is an important consideration when evaluating any new CT-based method for CAC quantification. Dual-energy CT generally requires a higher radiation dose compared to single-energy CT due to the acquisition of two datasets at different energy levels [28]. However, recent advancements in CT technology, such as photon-counting detectors, have the potential to reduce radiation dose while maintaining or even improving image quality [29].

Photon-counting CT is an emerging technology that utilizes energy-resolving detectors to discriminate between different energy levels of incoming photons. This technology enables the acquisition of multiple energy bins from a single exposure, eliminating the need for multiple acquisitions at different energy levels [30]. Photon-counting CT has the potential to improve material decomposition and reduce radiation dose compared to conventional dual-energy CT [31]. The application of the proposed dual-energy material decomposition method for CAC quantification using photon-counting CT could potentially lead to improved detection of low-density and microcalcifications while minimizing radiation exposure to patients.

Regarding the slice thickness for the proposed CAC quantification method, the simulation study utilized a slice thickness of 0.5 mm. While this slice thickness is not strictly required for the method to function, previous studies have demonstrated that using a decreased slice thickness of 0.5 mm can improve the accuracy of Agatston scoring [24, 25]. The use of a smaller slice thickness allows for better visualization and quantification of small and low-density calcifications, which may be particularly beneficial for the detection of early-stage atherosclerosis. However, it is important to note that the optimal slice thickness for the proposed dual-energy material decomposition method may require further investigation using more realistic CT data and should be balanced against potential increases in radiation dose.

Accurate identification of CAC at or near the detection threshold necessitates an estimation of the region of interest containing potential CAC. In this investigation, we employed a previously validated automatic segmentation method [18]. In clinical settings, estimates of the coronary artery centerline may be derived from non-contrast CT scans through automated methodologies, such as atlas-based methods [32, 33]. Emerging advancements in deep learning offer encouraging prospects for the automatic segmentation of cardiac anatomy. These techniques show potential in delineating coronary artery centerlines accurately in non-contrast CT scans by employing supervised learning strategies on patient images like those found in the OrCaScore dataset [34]. These coronary artery centerlines can subsequently facilitate the automated generation of region of interests for the quantification of coronary artery calcium mass.

A pivotal aspect of this process is the material decomposition approach, which is executed on a voxel-by-voxel basis. This technique significantly reduces the challenges traditionally associated with precise region of interest segmentation. One of the key strengths of material decomposition in dual-energy CT scans is its ability to accurately quantify calcium mass independent of the immediate background intensity surrounding the region of interest. This independence from background intensity contrasts the volume fraction method, where accurate region of interest extraction is critical and heavily reliant on the surrounding context in non-contrast CT scans. Consequently, material decomposition not only simplifies the process but can enhance the accuracy and reliability of CAC mass quantification, particularly in regions where traditional methods may struggle to differentiate between calcium and surrounding tissues

Given that microcalcifications may signal early-stage atherosclerosis [23], enhanced sensitivity could hold significant implications for the early detection and treatment of cardiovascular disease. More realistic CT data may further underscore the benefits of dual-energy material decomposition as a CAC quantification technique, illuminating the path for future technological advances in this field.

It’s also worth noting that although our study shows potential benefits with dual-energy material decomposition for CAC quantification, the availability of dual-energy CT scanners in clinical settings remains limited. As dual-energy CT scanners become more widely accessible, the adoption of these methods may become more feasible.

The study also has limitations due to the lack of realistic cardiac hardware and the effect of patient movement on image data, which can cause artifacts [35]. Metal artifacts, such as those caused by surgical clips or dental fillings, can be easily confused with calcium, leading to an overestimation of calcium scores [36]. Similarly, motion artifacts, caused by irregular heart rhythms or inadequate breath-holding, can also result in an overestimation of calcium scores [6]. Dual-energy CT has the potential to minimize the impact of these artifacts on CAC quantification. The material decomposition capabilities of dual-energy CT may help differentiate between calcium and metal artifacts, reducing the risk of overestimation. Additionally, combining dual-energy CT with techniques such as retrospective ECG gating could help mitigate motion artifacts. Future investigations should incorporate images influenced by typical hardware and motion artifacts, use more realistic CT data, and consider the effects of various reconstruction techniques on material decomposition-based CAC quantification. This will help assess the robustness of the proposed method in clinical settings and further elucidate the potential benefits of dual-energy CT in addressing these artifacts.

## Conclusion

This study demonstrates the potential of the dual-energy material decomposition technique as a viable method for improving the sensitivity of coronary artery calcification detection, particularly for low-density calcium and microcalcifications that are often overlooked by the conventional Agatston scoring method. Although the dual-energy material decomposition method demonstrated lower accuracy compared to the volume fraction calcium mass technique in this phantom study, we believe that this limitation can be overcome by exploring more advanced material decomposition techniques and validating the method using more realistic CT data. This new method may serve as a valuable tool in enhancing early-stage atherosclerosis detection and subsequently improving patient outcomes in cardiovascular disease.
